# Enhanced expression of activity‐regulated cytoskeleton‐associated protein in the medial prefrontal cortex is involved in working memory performance

**DOI:** 10.1002/kjm2.12832

**Published:** 2024-04-16

**Authors:** Tsan‐Ju Chen, Dean‐Chuan Wang, Pei‐Chun Liu, Hui‐Shan Hung, Tsung‐Lin Cheng

**Affiliations:** ^1^ Department of Physiology, School of Medicine, College of Medicine Kaohsiung Medical University Kaohsiung Taiwan; ^2^ Department of Medical Research Kaohsiung Medical University Hospital Kaohsiung Taiwan; ^3^ Department of Sports Medicine, College of Medicine Kaohsiung Medical University Kaohsiung Taiwan; ^4^ Orthopaedic Research Center Kaohsiung Medical University Kaohsiung Taiwan; ^5^ Regeneration Medicine and Cell Therapy Research Center Kaohsiung Medical University Kaohsiung Taiwan

**Keywords:** arc, mPFC, T‐maze, working memory

## Abstract

Working memory (WM) is a cognitive function important for guiding the on‐going or upcoming behavior. A memory‐related protein Arc (activity‐regulated cytoskeleton‐associated protein) is implicated in long‐term memory consolidation. Recent evidence further suggests the involvement of hippocampal Arc in spatial WM. The medial prefrontal cortex (mPFC) is a key brain region mediating WM. However, the role of mPFC Arc in WM is still uncertain. To investigate whether mPFC Arc protein is involved in WM performance, delayed non‐match to sample (DNMS) T‐maze task was performed in rats with or without blocking new synthesis of mPFC Arc. In DNMS task, a 10‐s or 30‐s delay between the sample run and the choice run was given to evaluate WM performance. To block new Arc protein synthesis during the DNMS task, Arc antisense oligodeoxynucleotides (ODNs) were injected to the bilateral mPFC. The results show that, in rats without surgery for cannula implantation and subsequent intracerebral injection of ODNs, WM was functioning well during the DNMS task with a delay of 10 s but not 30 s, which was accompanied with a significantly increased level of mPFC Arc protein, indicating a possible link between enhanced Arc protein expression and the performance of WM. After preventing the enhancement of mPFC Arc protein expression with Arc antisense ODNs, rat's WM performance was impaired. These findings support enhanced mPFC Arc protein expression playing a role during WM performance.

## 1 INTRODUCTION

Working memory (WM) is a cognitive function that can temporarily maintain and manipulate task‐relevant information to guide on‐going or upcoming behavior.^1^, ^2^ In humans, besides aging, WM deficits are also observed in many psychiatric and neurological disorders such as schizophrenia, attention‐deficit/hyperactivity disorder, autism, Alzheimer's disease, Parkinson's disease, and brain damage^2^; accordingly, identifying the mechanisms underlying WM is important to develop therapy strategies.

Previous studies including functional imaging in humans and neural activity recording in animals have demonstrated that a key brain region mediating WM is the prefrontal cortex (PFC).^1^ Lesion studies in rodents reveal that the medial PFC (mPFC) in such as prelimbic (PrL) and infralimbic (IL) areas is involved in WM.^3^, ^4^ In particular, behavioral observations in rats show that lesions of the PrL and IL impair their performance on WM tasks when delays are imposed.^5^, ^6^ Among various tasks containing delay periods, the delayed non‐match to sample (DNMS) T‐maze task is a frequently used way to measure WM in rodents,^6^, ^7^, ^8^ and is thereby adopted in this study.

Synaptic plasticity is the change of synaptic efficacy in response to use or disuse, where long‐lasting synaptic plasticity is a major cellular mechanism underlying learning and memory.^9^ In the brain, protein products of numerous immediate early genes are closely related to memory, and among these, activity‐regulated cytoskeleton‐associated protein (Arc), encoded by an effector immediate early gene *Arc* or *Arg3.1*, is a critical regulator of long‐lasting synaptic plasticity.^9^ The induction of *Arc* expression by neuronal activity^10^, ^11^ and behavioral training^12^, ^13^, ^14^ exhibits benefits whereas the inhibition of Arc protein synthesis^13^, ^15^ impairs the consolidation of synaptic plasticity and memory. Although Arc is strongly implicated in long‐term memory consolidation,^15^, ^16^ the role of Arc in WM lacks sufficient evidence. A recent study reported the involvement of hippocampal Arc in spatial WM in response to anthocyanin supplementation.^17^ As for the mPFC, synaptic plasticity such as long‐term potentiation has been demonstrated^18^, ^19^; additionally, the involvement of mPFC Arc in long‐term extinction memory is reported recently,^20^ although the role of mPFC Arc in WM remains indeterminate.

To test the hypothesis that enhanced expression of mPFC Arc is critical for performing WM, DNMS T‐maze task was used to evaluate the WM in rats, and the intracerebral injection of Arc antisense oligodeoxynucleotides (ODNs) was given to block the new synthesis of mPFC Arc protein during the DNMS T‐maze task. The results suggest that enhanced mPFC Arc protein expression is important for WM performance on the DNMS T‐maze task.

## 2 MATERIALS AND METHODS

### 2.1 Animals

Male Sprague–Dawley rats aged 10–11 weeks were purchased from BioLASCO Taiwan Co., Ltd. They were housed in cages in a temperature‐ and humidity‐controlled room with a 12‐h light/dark cycle. An accommodation period of at least 7 days was given to all rats with food and water provided ad libitum before the start of the experiment. Once the rats were aged 12 weeks, a restricted amount of chow (~90% of the normal intake) was given throughout the experiment to increase animals' motivation to run the T‐maze task.^21^ All efforts were made to minimize the suffering of the animals and the number used. All experimental procedures in animals were reviewed and approved by the Institutional Animal Care and Use Committee of Kaohsiung Medical University (IACUC Approval No.: 100087).

### 2.2 WM evaluation

#### 2.2.1 Apparatus

**FIGURE 1:**
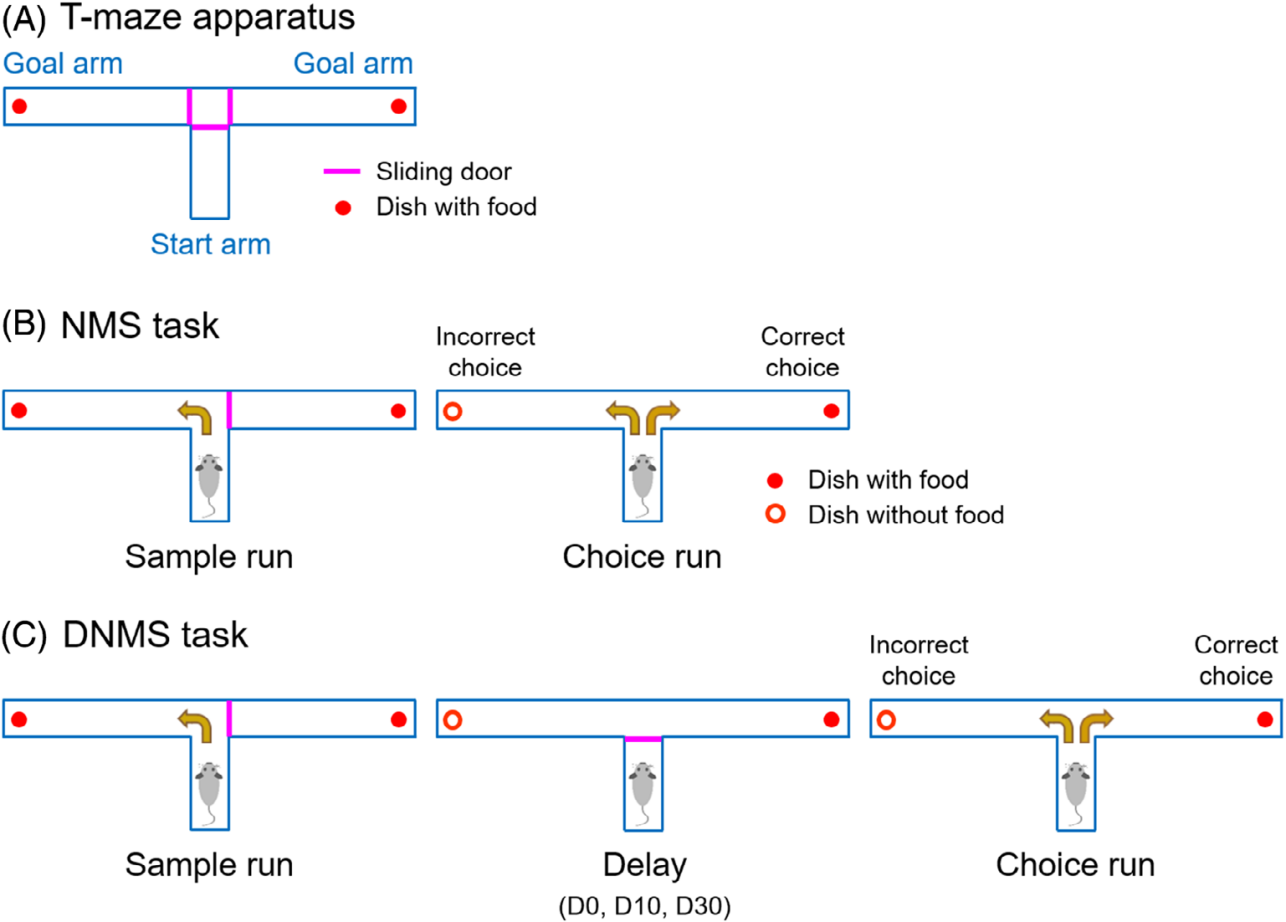
Evaluation of the WM in rats by a T‐maze task. (A) The T‐maze apparatus. (B), (C) The experimental procedure for performing one trial of NMS or DNMS task. DNMS, delayed non‐match to sample; D0, no delay; D10, a 10‐s delay; D30, a 30‐s delay; NMS, non‐match to sample; WM, working memory.

The T‐maze apparatus, composed of a start arm (30 cm in length; 12 cm in width; 15 cm in height) and two goal arms (60 cm in length; 12 cm in width; 15 cm in height), was made of black Plexiglas and equipped with three sliding doors to block the maze arms (Figure 1A). At the end of each goal arm, a small dish was secured to the floor of the maze using double‐sided tape to deliver odorless food pellets (0.2 g).

#### 2.2.2 Habituation

**FIGURE 2:**
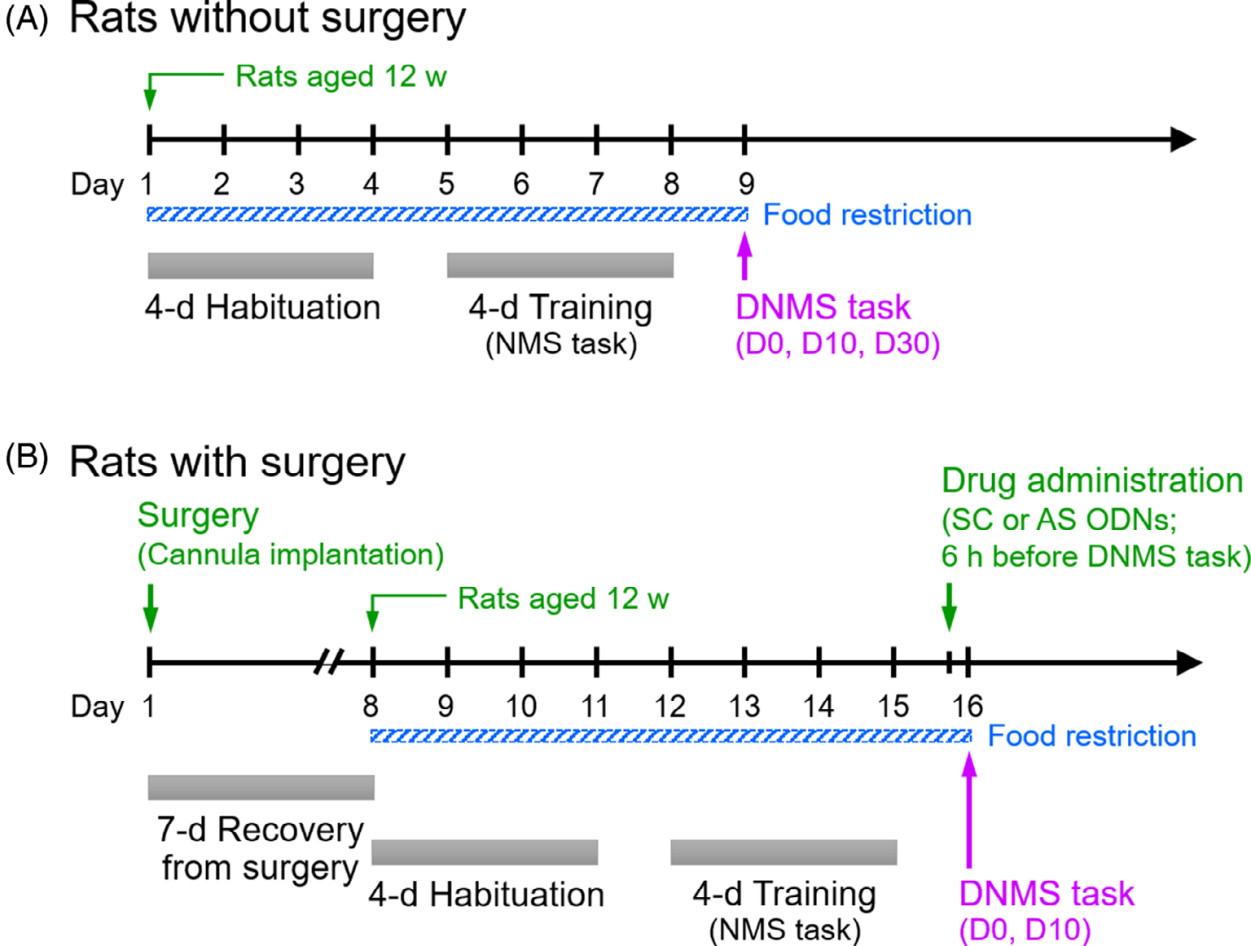
Experimental design and schedule. (A), (B) An experimental timeline for rats without surgery or with a surgery of cannula implantation. AS, antisense; ODNs, oligodeoxynucleotides; SC, scrambled.

The rats were habituated to the T‐maze apparatus for 4 days (five explorations/day) (Figure 2). Detailed processes are shown in the Supporting information.

#### 2.2.3 Training

After habituation, the rats were trained for 4 days to perform the non‐match to sample (NMS) task (10 trials/day) (Figures 1B and 2). Each trial consisted of two runs: a sample run in which the rat was forced to run to the open goal arm, and a choice run in which the rat must choose the previously unvisited goal arm (correct choice). A well‐trained rat was designated when its percentage of correct choices on the fourth training day was not less than 70% and higher than the percentage obtained on the first training day. Rats that did not fulfill this criterion were excluded from the study. Detailed training processes are shown in the Supporting information.

#### 2.2.4 DNMS T‐maze task

On the day following the last training day, the DNMS T‐maze task was performed to evaluate animal's WM (Figure 2). The above well‐trained rats were given 10 trials with a delay of 0 (for comparison), 10 or 30 s (denoted as D0, D10, and D30, respectively) between the sample run and the choice run (Figure 1C). The rats stayed in the start arm with the door closed during the period of delay. The percentages of correct choices serving as the behavioral performance of WM were calculated for statistical analysis.

### 2.3 Guide cannula implantation

**FIGURE 3:**
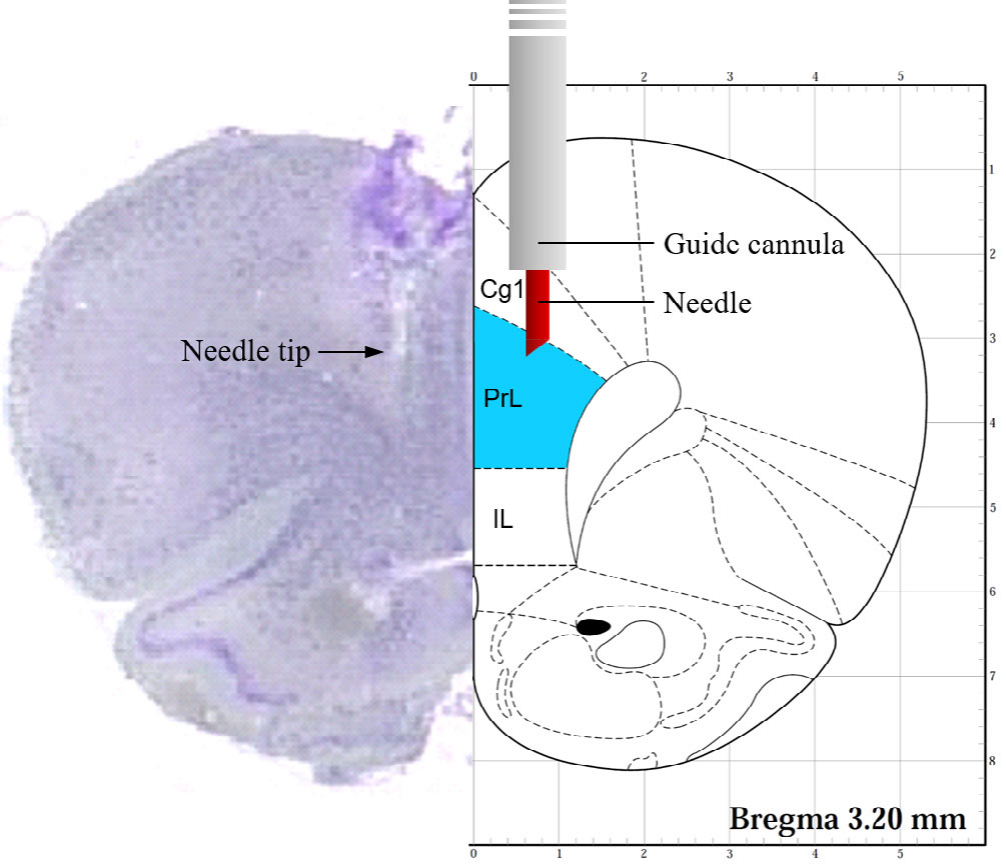
Illustration of the placement of cannula and needle in a coronal section of the rat brain. Right side: PrL (prelimbic cortex) is the target area to be treated. Left side: A coronal Nissl‐stained brain section (20‐μm thick) revealed that the needle tip is within the PrL. Cg1, cingulate cortex, area 1; IL, infralimbic cortex.

Two small holes were drilled over the bilateral mPFC (+3.2 mm anteroposterior, ±0.75 mm mediolateral from the bregma) according to the stereotaxic coordinate atlas.^22^ Then, two guide cannulas (12 mm in length, 0.7 mm in outer diameter) were implanted into the mPFC (−2.2 mm dorsoventral from the skull) (Figure 3). Finally, a stylet was inserted into the cannula to prevent obstruction. Rats were allowed to recover from surgery for 7 days before habituation to the T‐maze apparatus (Figure 2B). Detailed processes are shown in the Supporting information.

### 2.4 Drug administration

Antisense ODNs against *Arc* mRNA and scrambled ODNs serving as a control were designed as shown in the previous study.^23^ To inject ODNs into the mPFC, the stylet was removed and a needle connected to a 1 μL microsyringe was inserted through the guide cannula until its tip was 1.0 mm below the end of the cannula and targeting the prelimbic (PrL) area (Figure 3). A 0.5 μL solution containing Arc antisense ODNs (0.2 nmol) or scrambled ODNs (0.2 nmol) was injected for 30 s into the mPFC, and then the needle remained in place for 30 s. After that, the needle was removed and the stylet was inserted back into the guide cannula. The DNMS task was performed 6 h after drug injection (Figure 2B).

### 2.5 Western blot analysis

Thirty minutes after finishing the habituation session or the T‐maze task, the rats were sacrificed with CO_2_ inhalation. Animals' brains were immediately removed and the frontal cortices containing mPFC were cut into brain slices of 2 mm thickness. After that, the mPFC was isolated and homogenized in lysis buffer containing protease inhibitor. The subsequent processes for Western blot analysis are shown in the Supporting information. In this study, primary antibodies recognizing Arc (1:200) (Santa Cruz Biotechnology, USA) and actin (1:10,000) (Sigma‐Aldrich, USA) were used.

### 2.6 Statistical analysis

Statistical analysis was performed using IBM SPSS Statistics 20 software. Data are shown as mean ± SEM and presented by box plots. The percentages of correct choices during the 4‐d training session and the DNMS task were analyzed by two‐way mixed‐design ANOVA with Bonferroni's correction for multiple comparisons. Data obtained from other results were analyzed by Student's *t*‐test or one‐way ANOVA followed by post hoc Tukey HSD test as appropriate. The significance level was set at *p* < 0.05.

## 3 RESULTS

### 3.1 WM was maintained during the DNMS task with a 10‐s but not 30‐s delay

**FIGURE 4:**
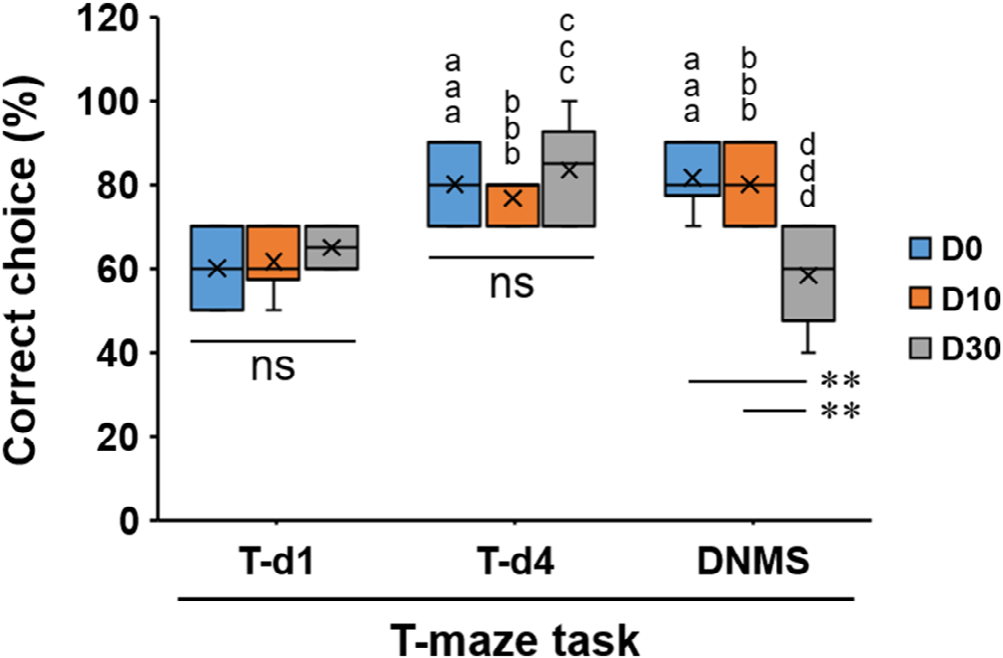
Percentages of correct choices in rats without surgery performing the T‐maze task. T‐d1: the first training day; T‐d4: the fourth training day; ^aaa^, ^bbb^, ^ccc^: *p* < 0.001 (vs. D0‐T‐d1, D10‐T‐d1, and D30‐T‐d1 respectively); ^ddd^: *p* < 0.001 (vs. D30‐T‐d4); **: *p* < 0.01; DNMS, delayed non‐match to sample; ns, not significant.

Well‐trained rats were divided randomly into D0, D10, and D30 groups (*n* = 6/group) to perform the DNMS task with a delay of 0, 10, or 30 s respectively, with the changes in percentages analyzed by two‐way mixed‐design ANOVA with GROUPs (D0, D10, D30) and DAYs of T‐maze task (T‐d1, T‐d4, DNMS) as independent and dependent factors, respectively (Figure 4; Figure S1). Because there was a significant interaction effect between GROUPs and DAYs (F(4,30) = 15.489, *p* < 0.001), the simple main effects test was then conducted. Statistical significances were found for the DAYs within each GROUP (F(2,30) = 27.069 (D0), 17.758 (D10), and 31.207 (D30); *p* < 0.001); additionally, a statistical significance was found for the DNMS task among three groups (F(2,45) = 13.197, *p* < 0.001). Multiple comparisons revealed that, in groups D0 and D10, the percentages were significantly increased (^aaa,bbb^: *p* < 0.001) on the fourth training day (T‐d4) and on the day for DNMS task (DNMS) when compared with those on the first training day (T‐d1); however, in group D30, the percentage was significantly increased (^ccc^: *p* < 0.001) on T‐d4 when compared with that on T‐d1, but on DNMS day, the percentage returned to a level not significantly different from that on T‐d1. Moreover, on DNMS day, the percentage for group D30 was significantly lower than those for groups D0 and D10 (**: *p* < 0.01). These results indicate that, on the DNMS day, well‐trained rats (D0) still remembered the rule for choice run, and furthermore, rat WM was maintained when the delay was 10 s (D10) whereas a 30‐s delay (D30) was too long to keep WM functioning well.

### 3.2 Levels of mPFC arc protein showed no significant change following 4‐d habituation and 4‐d training

The levels of mPFC Arc protein did not change significantly following the 4‐d habituation and 4‐d training sessions (Figure S2), indicating that the expressions of mPFC Arc protein remained at the basal levels when animals had become habituated to the T‐maze apparatus. Detailed statistical comparisons are shown in the Supporting information.

### 3.3 mPFC Arc protein expression was enhanced more in rats performing the DNMS T‐maze task with a 10‐s delay

**FIGURE 5:**
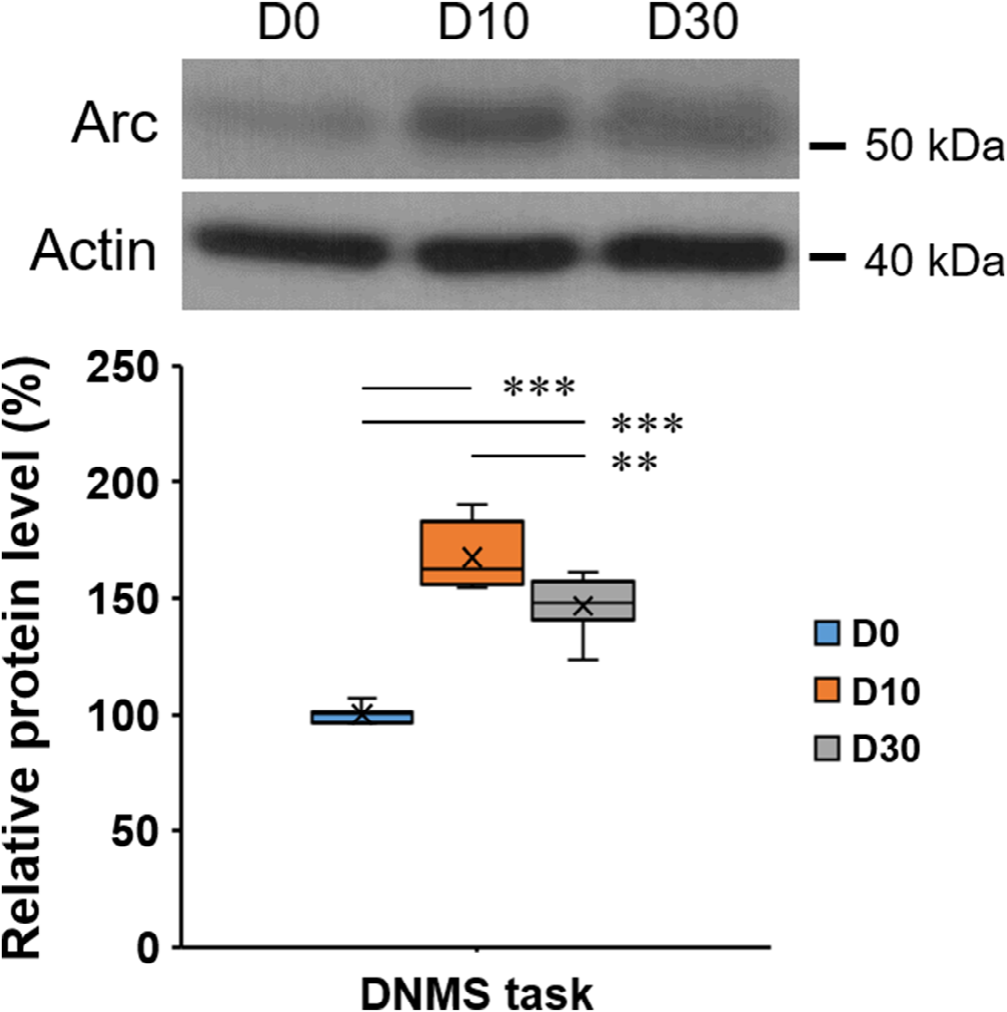
A higher enhancement of mPFC Arc protein expression in rats performing the DNMS task with a 10‐s delay. Actin was used as loading control. Protein levels in group D0 were normalized to 100%. DNMS, delayed non‐match to sample. **: *p* < 0.01, ***: *p* < 0.001.

To evaluate the change of mPFC Arc protein expression in rats performing the DNMS task, a comparison was made among D0, D10, and D30 groups. As shown in Figure 5, there is a significant difference among these three groups (one‐way ANOVA, F(2, 21) = 80.731, *p* < 0.001), with the post hoc test revealing that, when compared with the D0 group, significantly enhanced levels were found in both D10 and D30 groups; however, when compared with the D10 group, the D30 group enhanced Arc protein expression at a significantly lesser degree (**: *p* < 0.01), indicating a reduced efficiency of Arc induction in the D30 group.

### 3.4 The speeds of rats moving in the T‐maze did not change after the surgery of cannula implantation and the injection of Arc antisense ODNs

After the surgery of cannula implantation and the injection of Arc antisense ODNs, the moving speeds of rats in the T‐maze did not change significantly (Figure S3), indicating that animal's ability to explore the T‐maze and obtain food rewards was intact. Detailed measurement and statistical comparisons are shown in the Supporting information.

### 3.5 WM was impaired in Arc antisense ODN‐treated rats but not scrambled ODN‐treated rats

**FIGURE 6:**
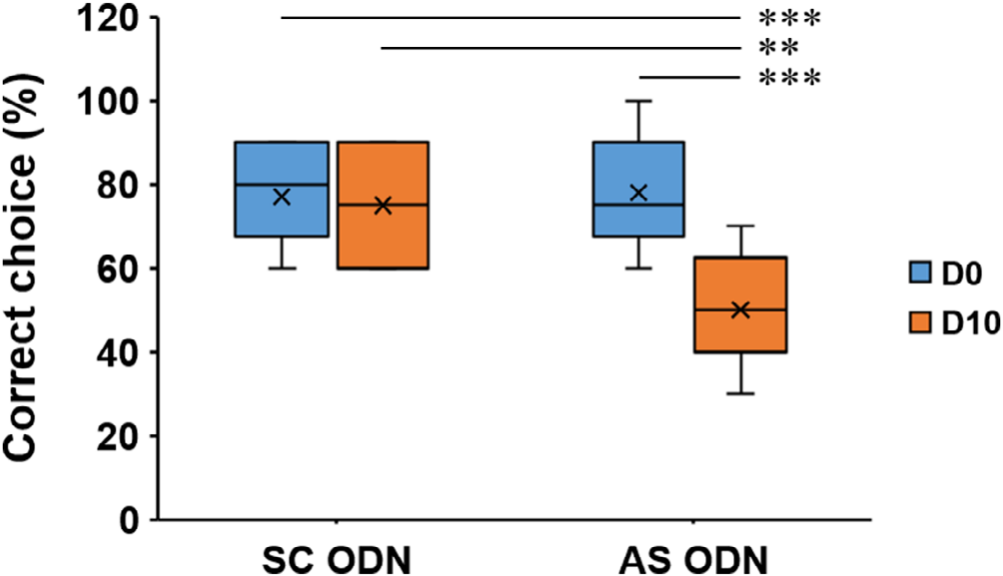
Reduced percentages of correct choices in Arc AS ODN‐treated rats performing the DNMS task with a 10‐s delay. DNMS, delayed non‐match to sample. **: *p* < 0.01, ***: *p* < 0.001.

To investigate the role of enhanced mPFC Arc protein in performing WM, scrambled (SC) or Arc antisense (AS) ODNs were injected to rats. Figure S4 depicts the cannula placement in the mPFC. According to the findings shown in Figure 4, DNMS task with a 10‐s delay was performed for the subsequent experiments as shown in Figure 2B where well‐trained rats were divided into four groups (*n* = 10/group) according to the treatments (SC or Arc AS ODNs) and the delay (D0 or D10) during the DNMS task. The results shown in Figure 6 revealed a statistically significant difference in the percentages of correct choices among these four groups (one‐way ANOVA, F(3,36) = 10.724, *p* < 0.001). The post hoc test revealed that, in SC ODN‐treated rats, no significant difference was found between the D0 and D10 groups, whereas in Arc AS ODN‐treated rats, a significantly reduced percentage was found in the D10 group (*p* < 0.001) (Figure 6). There was no significant difference between groups of D0‐SC ODN and D0‐AS ODN, indicating that Arc AS ODN‐treated rats also performed the T‐maze task with no delay normally; additionally, when comparing SC ODN‐treated rats (Figure 6) with rats not undergoing surgery (Figure 4), no significant difference was found (D0‐SC ODN vs. D0‐DNMS, D10‐SC ODN vs. D10‐DNMS, Student's *t*‐test, *p* > 0.05), indicating that the intracerebral injections of SC ODNs exerted no significant effects on WM performance. In contrast, the results for Arc AS ODN‐treated rats revealed that blocking new synthesis of mPFC Arc protein impairs WM performance on the DNMS task with a 10‐s delay.

### 3.6 Levels of mPFC Arc protein were not enhanced in Arc antisense ODN‐treated rats performing the DNMS T‐maze task with a 10‐s delay

**FIGURE 7:**
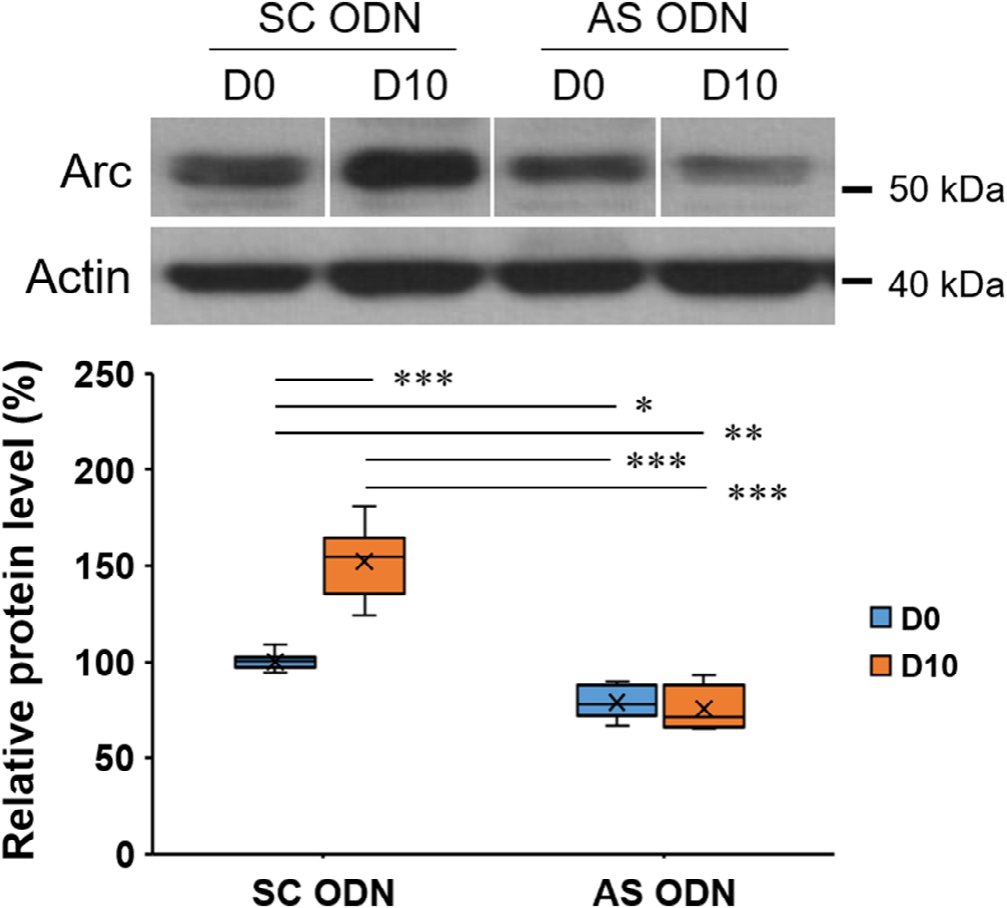
Failure of enhancing mPFC Arc protein expression when performing the DNMS task with a 10‐s delay in Arc AS ODN‐treated rats. Splicing was done for presentation purposes. Actin was used as loading control. Protein levels in group D0‐SC ODN were normalized to 100%. *: *p* < 0.05; **: *p* < 0.01; ***: *p* < 0.001.

As shown in Figure S5, in the absence of performing the DNMS task, Arc AS ODN‐treated rats exhibited significantly decreased basal Arc protein levels when compared with the SC ODN‐treated rats (*n* = 5/group, Student's *t*‐test, *p* < 0.001), indicating the success of blocking new synthesis of Arc protein. After that, the suppressed effect of Arc AS ODNs on the induction of mPFC Arc protein synthesis during the DNMS T‐maze task with a 10‐s delay was evaluated. A significant difference in mPFC Arc protein levels among the aforementioned four groups was found (one‐way ANOVA, F(3, 28) = 62.076, *p* < 0.001) (Figure 7). Post hoc testing further revealed that, in SC ODN‐treated rats, the levels of mPFC Arc protein were significantly enhanced in the D10 group when compared with the D0 group (*p* < 0.001). This finding is consistent with the result derived from rats without surgery (Figure 5), while in contrast, in Arc AS ODN‐treated rats, no significant difference was found between the D0 and D10 groups. These findings support the success of blocking enhanced expression of mPFC Arc protein by Arc AS ODNs when performing T‐maze task with a 10‐s delay.

## 4 DISCUSSION

In contrast to the long‐term memory in which synaptic plasticity is the most important neural mechanism, the mechanism underlying WM is still controversial. WM has long been regarded as solely dependent on persistent activity in the PFC^24^, ^25^; however, recent evidence suggests that activity‐silent dynamics in the PFC plays a role in WM and thus different forms of fast synaptic plasticity are proposed to support WM.^24^, ^25^ Whether Arc protein plays a role in WM‐related plasticity still lacks evidence.

Hippocampal Arc protein plays a role in long‐term memory and possibly also in WM. Feeding aged rats with anthocyanin‐rich extract for 6 weeks improves their spatial WM, which is accompanied with increased hippocampal Arc protein,^17^ although another recent study used mice and a modified spontaneous alternation behavior test in Y‐maze failed to confirm the involvement of hippocampal Arc in WM performances.^26^ On the other hand, mPFC is a key region mediating WM^1^, ^3^, ^4^; however, whether mPFC Arc plays a role in WM still lacks direct findings. In rats, a subcutaneous injection of α_7_ nicotinic acetylcholine receptor (nAChR) agonists increased the expression of Arc mRNA and Arc protein in the mPFC, suggesting that α_7_ nAChR agonist exerts its cognitive‐enhancing actions at least partly through activating brain regions critically involved in WM and attention.^27^, ^28^ Nevertheless, these studies did not evaluate animal's behavioral performance.

In Figure 4, rats performing the DNMS task with a 10‐s but not 30‐s delay maintained a significantly high percentage of correct choices. These findings indicate that rat's WM functions well within 10 s but fails with an extension to 30 s, which is consistent with the previous study^7^ and is in line with the concept that longer delays increase task difficulty and lead to poorer performance.^29^ Interestingly, although enhanced mPFC Arc protein expressions were observed in both D10‐DNMS and D30‐DNMS groups (Figure 5), a significantly less enhancement was found in D30‐DNMS group (vs. D10‐DNMS), indicating that a 30‐s delay might lead to a decrease in Arc induction. Because the DNMS T‐maze task is dependent on the mPFC,^7^, ^29^, ^30^ previous evidence has revealed a functional dissociation of mPFC; that is, vHPC (ventral hippocampus) inputs to the mPFC support spatial encoding (sample phase),^30^, ^31^ MD (the mediodorsal nucleus of the thalamus) inputs to the mPFC support the maintenance of working memory by stabilizing task‐relevant prefrontal activity (delay phase), and mPFC inputs to the MD supported the retrieval of memory for action execution (choice phase) (Figure S6).^30^ On the other hand, exposure to a novel environment for 5 min followed by a 30‐min rest increases Arc mRNA and protein expression in hippocampal and neocortical neurons^32^, ^33^; in particular, a significant increase in mPFC Arc mRNA was found.^33^ In the present study, the rats were exposed to a novel environment (T‐maze) initially, then following 4‐d habituation and 4‐d training, they were familiarized with the T‐maze and thus the levels of mPFC Arc protein remained at the basal level (Figure S2). Because the DNMS task was performed on the day following the last training day (T‐d4), the D0 group could be regarded as T‐d5 (training for 5 days) and thus the Arc protein level remained low (Figure 5). In contrast, it is reasonable to suggest that, in Figure 5, enhanced mPFC Arc protein expressions found in D10 and D30 groups were induced by the activity of the MD‐to‐mPFC pathway. In the D30 group, a lesser enhancement of Arc protein expression reflects decreased task‐relevant mPFC activity during the delay phase, and as a result, the activity of mPFC‐to‐MD projections that supports subsequent choice is reduced, leading to worse behavioral performance of working memory.

To further investigate the role of mPFC Arc, intracerebral injections of Arc AS ODNs were given to rats to block the induction of mPFC Arc protein synthesis during the DNMS task. In Figures 6 and 7, the results obtained from SC ODN‐treated rats were similar to those found in rats without surgery for cannula implantation (Figures 4 and 5). As for the Arc AS ODN‐treated rats, when compared with the D0‐SC ODN group, the D0‐AS ODN group showed a significant decrease in Arc protein level (Figure 7), which is similar to that found in rats without performing DNMS task (Figure S5). Most importantly, the enhancement of Arc protein expression shown in the D10‐SC ODN group was absent in the D10‐AS ODN group (Figure 7). These findings support the aforementioned suggestion that the MD‐to‐mPFC pathway is activated during the delay phase but not under the condition of no delay (D0) (Figure S6),^30^ which leads to the induction of mPFC Arc protein synthesis (enhanced expression) in the D10‐SC ODN group. In contrast, the enhancement of Arc protein expression failed in Arc AS ODN‐treated rats (D10‐AS ODN group); furthermore, in parallel with the finding shown in Figure 7, a significantly reduced percentage of correct choices was found in the D10‐AS ODN group (Figure 6), suggesting that the enhanced expression of mPFC Arc protein is important for WM performance on the DNMS T‐maze task with a 10‐s delay.

In conclusion, our findings support that enhanced expression of mPFC Arc protein plays an essential role in WM performance, and particularly, in rats, the DNMS T‐maze task with a 10‐s delay is suitable for evaluating the performance of WM and the changes of memory‐related molecules such as Arc protein in the mPFC, which could thus serve as a model for further investigations.

## CONFLICT OF INTEREST STATEMENT

The authors declare no conflict of interest.

## Supporting information


**DATA S1** Supporting information.
