# Supplementary material for: Enhanced expression of activity‐regulated cytoskeleton‐associated protein in the medial prefrontal cortex is involved in working memory performance
Source: Kaohsiung J Med Sci. 2024 Apr 16;40(6):553–60. doi: 10.1002/kjm2.12832 (PMC11895562; doi:10.1002/kjm2.12832)

Supporting information

- - 1. *Habituation*

The rats were handled for 3 consecutive days (10 min/day) to attenuate handling-induced stress during the following experiments. Then, they were habituated to the T-maze apparatus for 4 days (five explorations/day) (Figure 2). The rats were allowed to explore freely in the T-maze with all sliding doors open until they ate food pellets at the ends of both goal arms. After that, to start a new exploration, the rats were returned to the start arm with the sliding door closed for one min.

- - 1. *Training*

After habituation, the rats were trained for 4 days to perform the non-match to sample (NMS) task which is composed of ten trials per day (Figures 1B and 2). Each trial consisted of two runs: a sample run in which one goal arm was blocked so that the rats were forced to run to the open goal arm and rewarded with one food pellet at the end of arm, and a choice run followed immediately in which all blocks were removed. That is, after finishing the sample run, the rat was returned to the start arm to choose one goal arm to go into for obtaining the reward. All rats were trained to learn the rule that, in the choice run, a reward of one food pellet was given for choosing the previously unvisited arm (correct choice) and no reward was supplied for choosing the previously visited arm (incorrect choice). Left/right allocations for the sample and choice runs were pseudo-randomized over ten trials per day, with no more than three consecutive sample runs to the same side. The intertrial interval was one min during which the rats stayed in the start arm with the door closed. A well-trained rat was designated when its percentage of correct choices on the fourth training day was not less than 70% and higher than the percentage obtained on the first training day. Rats that did not fulfil this criterion were excluded from the study.

- 1. *Guide cannula implantation*

Rats were anesthetized with sodium pentobarbital (50 mg/kg, i.p.) and placed on a stereotaxic apparatus. After exposing the skull, two small holes (diameter: 0.8 mm) were drilled over the bilateral mPFC (coordinates: +3.2 mm anteroposterior, ± 0.75 mm mediolateral from the bregma) according to the stereotaxic coordinate atlas.^22^ Then, two stainless steel guide cannulas (12 mm in length, 0.7 mm in outer diameter) were implanted into the mPFC (coordinates: -2.2 mm dorsoventral from the skull) and fixed to the skull with stainless steel screws and dental cement (Figure 3). Finally, a stylet was inserted into the cannula to prevent obstruction. Rats were allowed to recover from surgery for 7 days before habituation to the T-maze apparatus (Figure 2B).

- 1. *Western blot analysis*

On the indicated days, 30 minutes after finishing the habituation session or the T-maze task, the rats were sacrificed with CO_2_ inhalation. To detect the protein levels in the mPFC, animals’ brains were immediately removed and the frontal cortices containing mPFC were cut into brain slices of 2 mm thickness. After that, the mPFC was isolated and homogenized in lysis buffer containing protease inhibitor. The homogenates were centrifuged at 13,000 ×g for 20 min at 4°C. Protein in the supernatant was quantified using a BCA protein assay kit (ThermoFisher, Waltham, MA, USA) and then thirty micrograms of protein were separated by 10% Bis-Tris gel (Invitrogen, Carlsbad, CA, USA) in MOPS running buffer (Invitrogen, Carlsbad, CA, USA). Subsequently, proteins were transferred to polyvinylidene difluoride (PVDF) membranes (Millipore, Burlington, MA, USA). Following a blocking step, the membranes were probed with primary antibodies against Arc protein (1:200) (Santa Cruz Biotechnology, CA, USA) and actin (1:10,000) (Sigma-Aldrich, St. Louis, MO, USA) overnight at 4°C. Then, after a washing step, the membranes were incubated with a horseradish peroxidase-conjugated secondary antibody (1:5,000) (Chemicon, Temecula, CA, USA) for 60 min at room temperature. The signals of immunolabeling were detected by enhanced chemiluminescence (Invitrogen, Carlsbad, CA, USA) and were exposed to X-ray film. Finally, in order to measure protein expression levels, the intensities of specific bands corresponding to the proteins of interest were quantified by densitometric analysis using Gel-Pro Analyzer software (Media Cybernetics, Rockville, MD, USA). In almost all protein samples, Western blot analysis was performed twice for better reliability.

*3.2. Levels of mPFC Arc protein showed no significant change following the periods of 4-d habituation and 4-d training.*

The changes of mPFC Arc protein expression following the sessions of 4-d habituation and 4-d training were evaluated by Western blot analysis (Figure S2). No significant difference of Arc protein levels was found among the four groups of rats, including one group always staying in their home cage (Cage, 100.00 ± 4.64 %), and three groups going through 4-d habituation (H-d4, 98.89 ± 2.09 %) and the following 1-d (T-d1, 101.20 ± 4.15 %) or 4-d (T-d4, 95.65 ± 3.49 %) training (one-way ANOVA, F(3, 28) = 0.410, p = 0.747). These results indicate that the expressions of mPFC Arc protein remained at the basal levels when animals had habituated to the T-maze apparatus.

*3.4. The speeds of rats moving in the T-maze did not change after the surgery of cannula implantation and the injection of Arc antisense ODNs.*

To evaluate whether the surgery of cannula implantation and/or the intracerebral injection of Arc antisense ODNs impair animal’s ability to explore the T-maze and obtain food rewards, the moving speeds of rats were measured after 7 days of recovery from the surgery and after 6 h of ODNs injection (Figure S3). The time spent in the sample run for obtaining the food reward was recorded to calculate the speed of the rat. Eight rats had no surgery (0.31 ± 0.02 m/s), 12 rats underwent surgery (0.29 ± 0.01 m/s), 9 rats had scrambled ODNs injection (0.31 ± 0.02 m/s) and 9 rats had Arc antisense ODNs injection (0.31 ± 0.02 m/s). No statistically significant difference in the speeds (Student’s *t*-test, p > 0.05) was found, indicating that both surgery and intracerebral injection of Arc antisense ODNs exerted no effect on animal’s moving ability and their motivation for performing the T-maze task.

**Figure S1.** The percentages of correct choices on the first training day (T-d1), the fourth training day (T-d4) and the following day for performing DNMS task (DNMS).


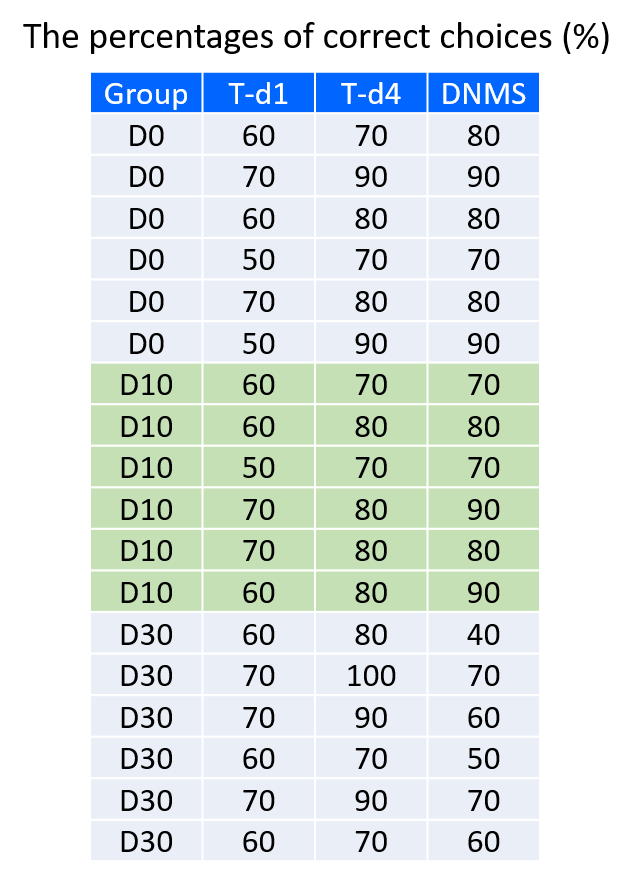


**Figure S2.** No significant change in levels of mPFC Arc protein following 4-d habituation and 4-d training sessions. Actin was used as loading control. Protein levels of Arc in rats always staying in their home cage (Cage) were normalized to 100%. H-d4: habituation for 4 days; T-d1, T-d4: training for 1 day or 4 days.


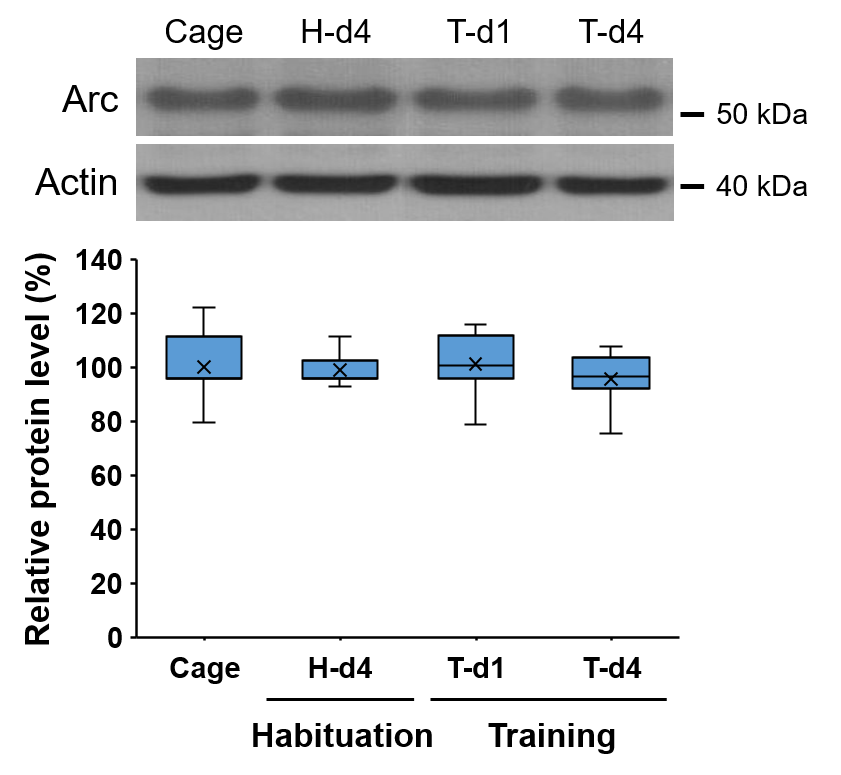


**Figure S3.** No change in the moving speeds of rats followed bilateral implantations of cannulas and injections of Arc antisense (AS) ODNs. **(A)** After 7 days of recovery from the surgery of cannula implantation, the moving speeds of rats showed no statistically significant change when compared with those of the rats without any surgery. **(B)** After 6 h of ODNs injection, the moving speeds of rats showed no statistically significant difference between two groups of rats treated with scrambled (SC) ODNs and Arc antisense (AS) ODNs respectively. ns: not significant.


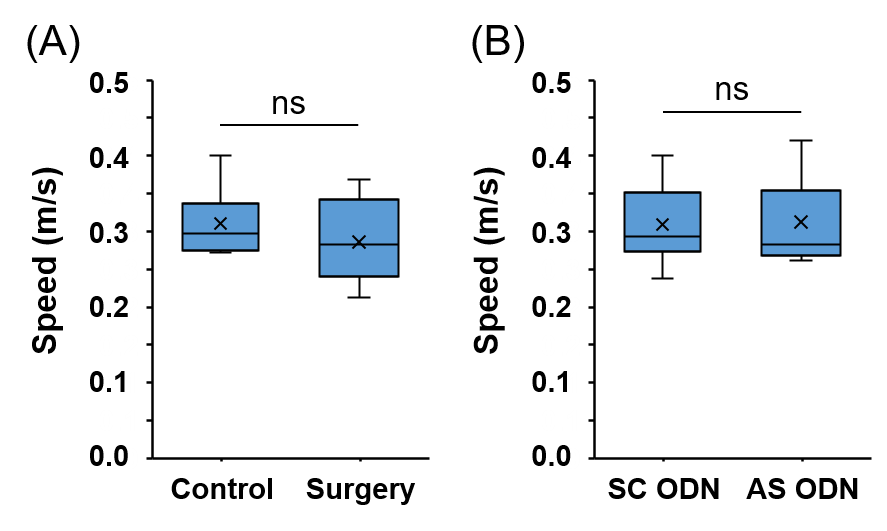


**Figure S4.** Cannula placement in bilateral mPFC. Schematic diagrams show mPFC cannulae placement relative to bregma. Blue dots indicate the sites of the needle tips as shown in Figure 3. For clarity, only the placement of D10-AS ODN group is shown.


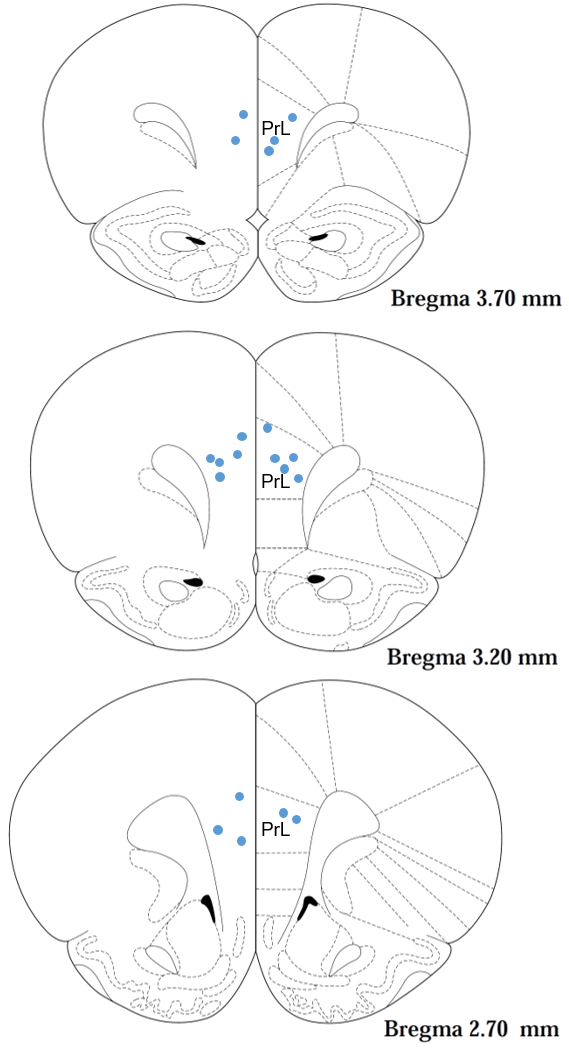


**Figure S5.** The expression of mPFC Arc protein in SC ODN- and Arc AS ODN-treated rats without performing the DNMS task. A significant decrease was found after treating rats with Arc AS ODNs. ***: p < 0.001.


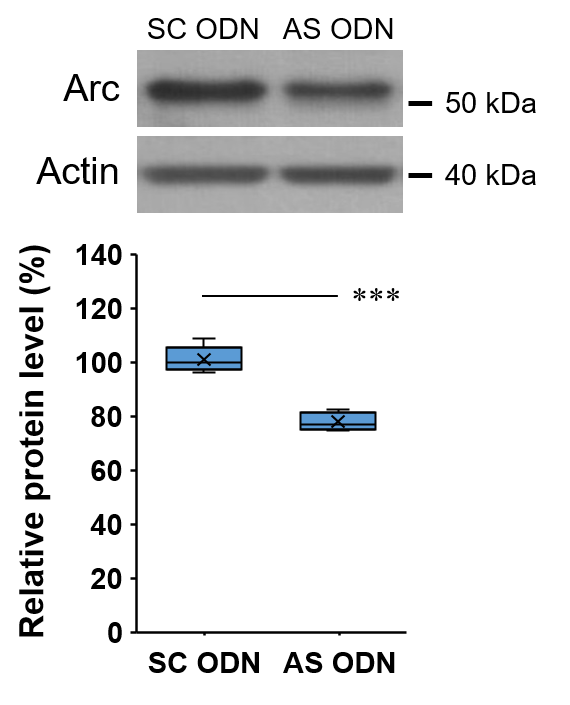


**Figure S6.** Illustration of the functional dissociation of mPFC for different phases in the DNMS task. According to the previous study,^30^ the vHPC-to-mPFC, MD-to-mPFC and mPFC-to-MD pathways are activated during the sample, delay and choice phases respectively. MD: mediodorsal thalamus; mPFC: medial prefrontal cortex; vHPC: ventral hippocampus.


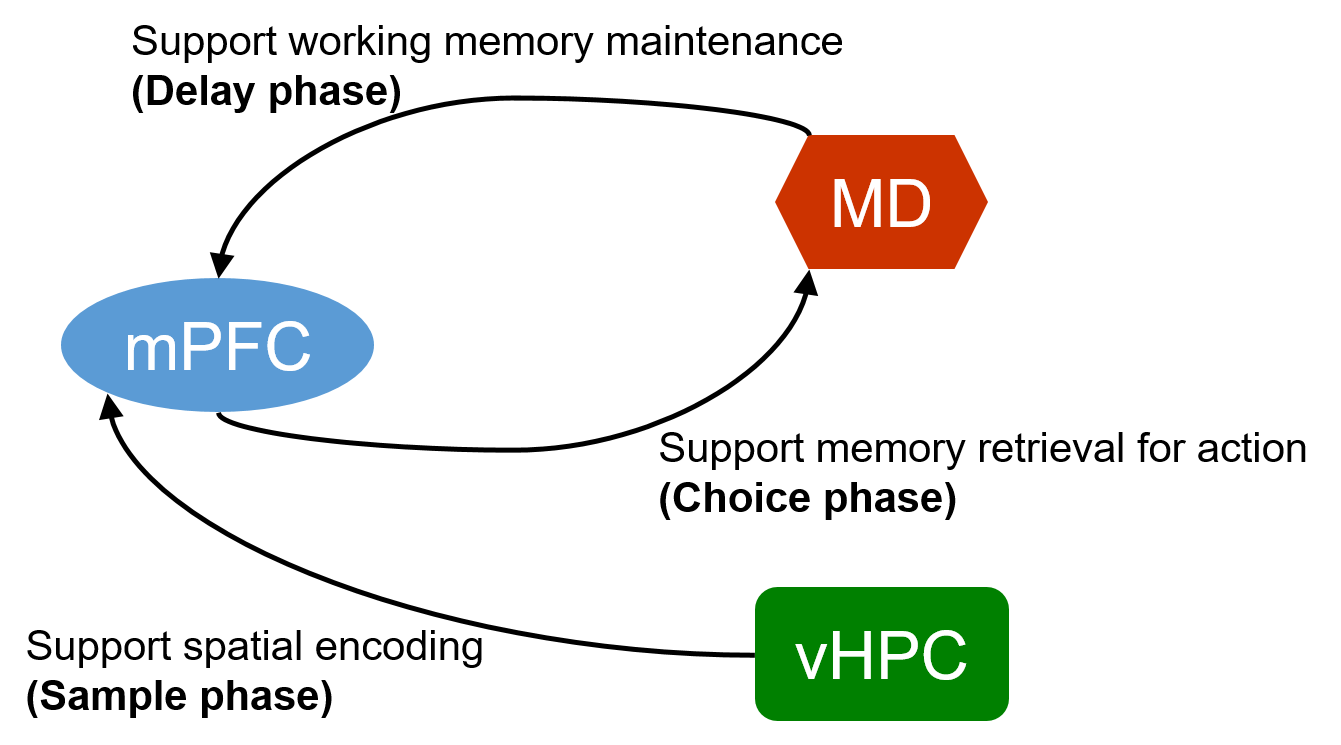

Supplement: Supplementary file 1 — DATA S1 Supporting information. [file KJM2-40-553-s001.doc]
